# Does adoption of new technologies require high operative volume? Our results with sutureless aortic bioprostheses

**DOI:** 10.1186/1749-8090-10-S1-A296

**Published:** 2015-12-16

**Authors:** Burak Can Depboylu, Patrick O Myers, Parmeseeven Mootoosamy, Jalal Jolou, Dominique Vala, Saziye Karaca, Marc Licker, Afksendiyos Kalangos, Mustafa Cikirikcioglu

**Affiliations:** 1 Division of Cardiovascular Surgery, University Hospitals and Medical Faculty of Geneva, Geneva, Geneva, 1211, Switzerland; 2 Division of Anesthesiology, University Hospitals and Medical Faculty of Geneva, Geneva, Geneva, 1211, Switzerland

## Background/Introduction

In order to develop a new surgical program based on recently developed technologies, companies often require a minimal volume of operations. Sutureless aortic bioprostheses were recently introduced in order to make the operation simpler, faster and more user friendly.

## Aims/Objectives

The aim of this study is to review our results with the Edwards Intuity Elite sutureless bioprosthetic aortic valve.

## Methods

The perioperative data of patients who underwent aortic valve replacement during the last year by sutureless bioprotheses (Edwards Intuity Elite, n = 7) were reviewed retrospectively and compared to those who received a conventional bioprostheses (Edwards Perimount Magna Ease, n = 7).

## Results

Patients in the Intuity group were significantly older (76 vs. 64 years), but didn't differ significantly with regards to EuroSCORE-II or comorbidities. The operative times didn't differ significantly between groups, even though more patients in the Intuity group had concomitant procedures. No valvular and paravalvular leak or heart block were seen after the operation in both groups. Despite the median valve size being smaller in the Intuity group (21 vs. 25 mm), the postoperative gradients were significantly lower.

## Conclusion

New sutureless aortic bioprostheses were safe and effective for the surgical treatment of severe aortic stenosis and provided better hemodynamic results. During this intial learning curve, operative times didn't differ between groups, no per- or post-operative complications were observed. The adoption of new technologies doesn't require high volume, provided it is conducted by the same familiar team in a step-by-step way.

**Table 1:** 

		Intuity group (n = 7)		Conventional group (n = 7)		*p
			
		Median	Min.-Max.	Median	Min.-Max.	
**Age (years)**		76	71-79	64	43-82	**0.035**

**Euroscore II**		6.48	2.56-10.55	3.41	1.53-8.97	>0.05

**CBP time (min)**		102	58-218	102	60-158	>0.05

**Crossclamp time (min)**		78	45-133	79	52-118	>0.05

**Valve size**		21	21-27	25	23-27	>0.05

**Concomitant procedures**		4		2		

**Aortic Valve Gradients (mmHg)**	**Preop Max.**	71	30-95	49	28-106	>0.05
	
	**Preop Mean**	46.50	16-61	29.50	21-51	>0.05
	
	**Postop Max.**	15.50	8-19	26	18-47	**0.006**
	
	**Postop Mean**	9	7-13	14	9-24	**0.046**

